# Integrating Self-Determination Theory and Continuous Glucose Monitoring: Promoting Youth Development Among Campers with Type 1 Diabetes †

**DOI:** 10.3390/bs16010024

**Published:** 2025-12-22

**Authors:** Eddie Hill, Bethany Arrington, Taylor Harvey, Alexis Barmoh, Rowan Williams, Laura Hill

**Affiliations:** 1 Dumke College of Health Professions, Weber State University, Ogden, UT 84408, USA; laurahill2@weber.edu; 2 Department of Sport and Recreation Professions, Virginia Wesleyan University, Virginia Beach, VA 23455, USA; bcarrington@vwu.edu; 3 Department of Human Movement Sciences, Old Dominion University, Norfolk, VA 23529, USA; tharv002@odu.edu (T.H.); alexis.barmoh@gmail.com (A.B.); rhwillia@odu.edu (R.W.)

**Keywords:** type 1 diabetes, time in range, self-determination theory, multi-methods, blood glucose levels, diabetes camp

## Abstract

The purpose of this cohort study was to evaluate participants’ general self-management and experiences of autonomy while attending diabetes camp using quantitative and qualitative data collection. Through a partnership, an outdoor diabetes camp was designed to assist youth with type 1 diabetes (T1D) in their management. The REACH Teen program conducted a week-long summer camp for youth with T1D. The study was designed through Outcome-Focused Programming grounded in Self-Determination Theory (SDT) to meet campers’ needs of autonomy, competence, and relatedness. Campers participated in outdoor activities and diabetes education designed to increase healthy behaviors. Twenty-three campers completed a 24-item pre- and post-camp questionnaire measuring participants’ perceived levels of satisfaction or frustration of their three basic psychological needs. At the conclusion of camp, 21 youth participated in 35-min focus group interviews. Through a paired-sample *t*-test, all three measures were trending in a positive direction, with relatedness (R) being the closest to significance. Cloud-based biometric data was used to compute the percentage of TIR for the week, during camp hours. The results from the focus group interviews revealed three themes: lack of outside understanding, varying levels of autonomy, and experiences at REACH. Not reporting TIR data outside of camp was a limitation of this study. Diabetes medical specialty camps grounded in SDT can provide an opportunity for campers to internalize healthy behaviors needed to manage their diabetes.

## 1. Introduction

Type 1 diabetes (T1D) is a chronic autoimmune disorder characterized by immune-mediated destruction of pancreatic beta cells, leading to absolute insulin deficiency and dysregulated glucose homeostasis, and it is one of the most common chronic illnesses in youth (Imperatore et al., 2018). According to the American Diabetes Association, more than 24,000 children in the USA are diagnosed with T1D every year. This is a disease that requires 24/7 management (American Diabetes Association [ADA], 2022). T1D is managed by checking blood glucose levels, giving insulin, monitoring food, and making other constant daily decisions. Optimal glucose control helps to reduce the risk of chronic complications (Mustonen et al., 2022). One way to measure diabetes management is through tracking time in range (TIR), which is the quantity of time spent in the target blood glucose (blood sugar) range, which is between 70 and 180 mg/dL for most people (American Diabetes Association [ADA], 2023b). Time in range is important to consider because diabetes complications are less likely with more time spent in range (American Diabetes Association [ADA], 2023b). Medical advances in diabetes care, like continuous glucose monitors (CGMs) to read glucose levels, have arguably made opportunities to participate in outdoor recreation/recreation-focused settings more accessible while maintaining recommended TIR. The CGM, usually attached to the arm, is a small sensor that measures glucose in interstitial fluid. These devices track blood sugar in real-time, reducing the number of finger stick tests needed. These assist individuals with T1D by providing real-time data on how activity and diet impact glucose levels.

Worldwide, the number of people living with T1D is expected to double by 2040 (G. A. Gregory et al., 2022). When poorly managed, elevated blood glucose levels increase, which can lead to severe complications and can even be fatal. T1D is currently the 8th leading cause of death by disease in the United States, and “long-term complications include coronary heart disease, stroke, retinopathy, nephropathy, neuropathy, periodontal disease, and amputation of the lower extremity” (American Diabetes Association [ADA], 2020). Moreover, the life expectancy gap is 10 to 12 years between people with T1D and the general population. The advancement of medical devices, like CGMs, and the use of cloud-based technology, can be an effective tool to increase quality of life. Cloud-based technology allows for blood glucose levels to be automatically uploaded to a secure server. Using cloud-based technology at diabetes camp has demonstrated effectiveness at keeping youth within the recommended TIR (Blaylock et al., 2025).

Glycated hemoglobin (HbA1c) has been found to be greater during adolescence than at any other life stage (Hagger et al., 2018), with only 17% of youth reaching their target blood sugar (Allen et al., 2021). Proper management is vital in decreasing the risk of potential complications; however, a significant challenge of living with diabetes is carrying out proper self-care (Hill et al., 2019). Research suggests the added layer of responsibility is a key part of poor management among adolescents (Blaylock et al., 2025; Hill et al., 2025). It is important to explore the role of youth programs to address psychosocial needs and promote self-motivated behavior related to diabetes management for youth living with T1D (Allen et al., 2021).

It is vital for these youth to have a community of supporters to help reach these target blood sugar values. One way to offer support to youth with T1D is through community partnerships and through medical specialty camps. Organizations supporting diabetes (e.g., American Diabetes Association) suggest that camp positively impacts participant outcomes, including knowledge of diabetes management, confidence to manage diabetes, and emotional well-being (American Diabetes Association [ADA], 2023a). Furthermore, medical specialty camps, when strategically designed, could potentially influence the youth’s ability to better control their glycemic level (Blaylock et al., 2025). Many other benefits from a diabetes camp can be achieved, such as developing friendships with others who have a shared experience, being part of a community, and engaging with peers (Arrington et al., 2022).

Diabetes camps historically have served as critical experiences for youth with T1D, helping them connect with others and learning management strategies (John, 1946). The first diabetes camp was established less than five years after the discovery of insulin, significantly improving the lives of youth with T1D (Maslow & Lobato, 2009). More recent studies of diabetes camps have demonstrated psychosocial outcomes, such as satisfaction and attitude (Bultas et al., 2016), glycemic control (Kietaibl et al., 2024), and resiliency (Collins et al., 2021), as well as time in range (Nagl et al., 2022). Other research determined themes such as motivation-centered environment, skill practice, and perceptions of wellness through interviewing campers (Mattsson et al., 2021). Mattsson et al. (2021) further identify that mastering self-management is only one part of the camp experience, noting that connecting with others is also critical. Other studies have explored friendship at diabetes camp (e.g., Hill et al., 2025, parent perspectives and family diabetes camp (Haegele et al., 2022), and overall wellness at diabetes camp (Collins et al., 2021). However, very little research exists using theory-based programming (i.e., Self-Determination Theory) to guide cloud-based glycemic data collection and psychosocial outcomes combined with semi-structured interviews to determine the camp experience impact among youth.

### 1.1. Self-Determination Theory

Self-Determination Theory (SDT) is a theory of human motivation and wellness that has evolved and developed over numerous decades (Ryan et al., 2021). It is a meta-theory that includes six mini-theories. These include Cognitive Evaluation Theory, Organismic Integration Theory, Causality Orientations Theory, Basic Psychological Needs Theory, Goal Content Theory, and Relationships Motivation Theory (Deci & Ryan, 2012; Ryan & Deci, 2019). Self-Determination Theory views motivation as a continuum ranging from amotivation on one side of the scale and intrinsic motivation on the other side (Ryan & Deci, 2020). SDT revolves around three main components, which are a person’s basic psychological needs of autonomy, competence, and relatedness. Ryan and Deci (2020) define the three basic psychological needs as autonomy, a sense of initiative and ownership in one’s actions, competence, which focuses on learning and excelling, and relatedness, which is associated with being cared for and caring for others. When considering autonomy, the environment in which SDT is being applied needs to be considered. Specifically, for an environment to be autonomy supportive, the literature suggests that three areas are needed: provision of choice, perspective taking, and rational giving (Soe et al., 2025). The theory posits that when an individual’s basic psychological needs of autonomy, competence, and relatedness are met, individuals are better able to internalize healthy behaviors (Ryan & Deci, 2020).

SDT recognizes that people grow and develop and does not view motivation as linear. A meta-analysis investigating SDT applied to health determined that meeting these psychological needs helped to motivate long-term maintenance of health behaviors (Ng et al., 2012). Most youth with T1D recognize and comprehend why it is important to manage their diabetes properly and the potential complications that could arise if not managed well; thus, the motivating factors that drive youth to manage their diabetes should be considered (Blaylock et al., 2025). Due to how T1D is managed, youth might be challenged for intrinsic motivation to manage their diabetes, but they can move along the scale of motivation, reaching a level of integration (Arrington et al., 2022). When beginning to transition to more independent management, youth may start managing their diabetes through extrinsic motivation, but as they continue into adolescence and young adulthood, it is important that they internalize healthy behaviors needed to move across the motivation continuum (e.g., Haegele et al., 2022). Historically, the research on youth with T1D and programs grounded in SDT has been promising (e.g., Hill & Sibthorp, 2006). Additionally, studies have explored the parenting of youth with T1D, as it relates to autonomy supportive environments (Barmoh et al., 2024). These nearly 20 years of work in SDT and the youth with T1D space continue to grow and identify new approaches (e.g., CGM) to address motivation for diabetes management among youth.

### 1.2. Outcome-Focused Programming

The use of an Outcome-Focused Programming (OFP) model begins with selecting and prioritizing the desired outcomes of the camp (Hill et al., 2016). OFP uses specific recreation experiences that are engineered to meet the needs of individuals. The structure of the camp is then intentionally designed with these outcomes in mind. The reason for using an OFP model is that it ensures that all activities and programs within the camp are being designed and implemented with the set purpose of meeting certain goals or outcomes. OFP uses four steps. (1) Program goals should be identified and meaningful to the agency, the participants, and other partners. (2) There should be intentionally structured, theory-based program components to address the stated goals. (3) The desired goals must be assessed. (4) An organization should be required to publicize its outcomes (Hill et al., 2016). The motivation-focused theory, SDT, offers a framework for both scholars and practitioners to promote the internalization of healthy behavior through intentionally planned camp experiences so that well-positioned practitioners can execute step 2 in OFP (i.e., theory-based outcomes). By grounding the outcomes in theory, practitioners and scholars can collaborate to effectively measure the intended outcomes. Step 2 in OFP is critical in the remaining steps, especially for evaluating the goals. A structured camp experience focused on diabetes competence, for example, appropriately grounds the camp in SDT.

Past research suggests that SDT is a workable conceptual framework to study antecedents and outcomes of motivation for health-related behaviors (Ng et al., 2012). Similar to other medical specialty camps grounded in SDT, this theoretical framework assists in the development of outcomes related to autonomy, competence, and relatedness (Collins et al., 2021). Programming that is provided within an autonomy supportive environment while emphasizing opportunities to build competence, autonomy, and relatedness can promote positive health behaviors (Allen et al., 2021; Arrington et al., 2022; Deci & Ryan, 2012; Taylor et al., 2012). Overall, the transfer of skills relevant to managing diabetes, as well as enhancing quality of life, is facilitated using a theoretical framework for medical specialty camps. As T1D is expected to double worldwide by 2040 and there is no cure, medical specialty camps have an opportunity to impact quality of life. This study could serve as a model for other diabetes camp programs that want to structure theory-based outcomes while being tied to cloud-based technology for biometric measures. Using the OFP model, this approach could be scalable to increase sample sizes, resulting in robust statistics to structure programs that more effectively serve our youth. We posit that using this theory-driven programming model will positively impact youth with T1D. Using quantitative and qualitative data collection, the purpose of this cohort study was to evaluate participants’ general self-management and experiences of autonomy while attending a diabetes camp.

## 2. Materials and Methods

Recreate, Educate, Advocate, and Climb Higher (REACH) Teen is a medical specialty camp for youth ages 11–17 with T1D. The REACH Teen camp program provides youth with T1D opportunities to engage in outdoor recreation while also learning about diabetes and ways in which it can be managed. After piloting a two-day teen camp in the summer of 2021, REACH Teen was launched, a teen diabetes five-day camp in August 2022. Data from the 2021 study supported using specific camp activities and their expected effects on self-determination outcomes (Harvey et al., 2022). This five-day camp was designed specifically for youth with T1D. Each day of camp offered different activities and programs, but every day included a variety of outdoor activities, swimming, rock climbing, arts and crafts, STEM activities, and diabetes education. Using OFP grounded in SDT, each activity of the program was strategically designed to meet the three different dimensions of autonomy, competence, and relatedness. Additionally, provision of choice, perspective taking, and rationale giving were used by staff to create an autonomous, supported environment. Choice provision, at the practitioner’s discretion, helps to empower the participant and allows some ownership to the individual making the decision. Perspective taking often requires a shift in thinking, considering the view of the participant, in this case, the camper. At times, choice is limited. Campers need to treat lows; that is not a choice. When decisions are made, helping to give a rationale for suggestions is important so the individual can avoid the feeling of being told what to do. Additionally, providing a rationale also helps the campers make educated decisions. Overall, each activity was intentionally programmed and implemented to foster autonomy, competence, and relatedness (see Table 1).

### 2.1. Participants and Setting

This was an exploratory study using a pretest–posttest design with no control group, which was appropriate since we were not looking at causal relationships but rather evaluating the camp impact. The camp included 23 campers who ranged from 9 to 17 years old with T1D. Although the camp was designed for youth ages 11 to 17, campers under that age range were accepted to camp due to the lack of diabetes-specific programs accessible to them. Twenty-three participants had T1D, while five additional campers were siblings or friends of the T1D campers (for a total of twenty-eight youth at camp). The five siblings/friends were not surveyed. Using a camp on campus model, the five-day camp took place on the campus of a Mid-Atlantic university. Campers were exposed to a variety of campus facilities and amenities such as the swimming pool, rock climbing wall, challenge course, walking/running path, turf field, basketball court, and dining hall.

### 2.2. Data Collection

After Institutional Review Board approval was granted, consent forms were shared with parents as campers were registered for a diabetes camp. Methods are reported in accordance with a STROBE cohort study. Data collection for this cohort study was completed prior to, during, and at the conclusion of camp. Both prior to attending camp and at the conclusion of camp, campers had the opportunity to complete the Basic Psychological Need Satisfaction Frustration Scale (BPNSFS). Throughout the entirety of camp, biometric data was collected on campers’ blood glucose levels through the use of campers’ continuous glucose monitor (CGM). Qualitative data were collected through semi-structured focus groups at the conclusion of camp. Using a multi-method approach (i.e., questionnaires, interviews, and biometric data) provided the researchers with a more comprehensive understanding of the program’s impact on diabetes management.

### 2.3. Basic Psychological Needs Satisfaction Frustration Scale (BPNSFS)

To measure the dimensions of the SDT, campers completed the English version of the 24-item BPNSFS. The original scale has been formally validated in four culturally diverse samples: Peru, China, Belgium, and the United States (Van der Kaap-Deeder et al., 2020). Reliability coefficients were as follows: autonomy α = 0.87, relatedness α = 0.81, and competence α = 0.86. The BPNSFS is grounded in the Basic Psychological Need Theory, which is one of the six sub-theories within SDT and is composed of 24 total quantitative questions. The questions within the scale relate to frustration or satisfaction of the three psychological needs: autonomy, relatedness, and competence. An example question is “I feel free to choose which activities I do.” The 24 items were measured on a 5-point scale, with 1 = completely not true and 5 = completely true. This scale was administered as a paper copy both prior to starting camp and at the conclusion of camp. A paired-sample *t*-test was used to measure the potential change in the BPNSFS, and Cohen’s *d* was used for power.

### 2.4. Biometric Data

Researchers collected biometric data (time in range) through CampViews iOS version 1.9.2, a new software designed for diabetes camps. The innovation eliminates the need for traditional electronic medical records (EMRs) by creating a real-time tracking, communications, and research tool for the management of a chronic illness, such as diabetes. This platform allows cloud diabetes technologies to be interconnected in order for cohesive pre-, during, and post-care monitoring and collaboration. CampViews is an effective system, not only for healthcare professionals but also for the environments leveraging the technology that care for the ultimate end user, those with diabetes. CampViews is able to standardize care, increase awareness of glucose levels in real-time, and keep everyone on the same page. However, CampViews has yet to be validated. 

CampViews allows participants to sync their Dexcom, which is a CGM, giving camp administrators and medical staff access to their blood glucose levels in real-time. The medical director was on site and conversed with campers to provide them the ability to make choices and to monitor these choices for safety. Continuous glucose monitors help people living with T1D manage their glucose levels and track their time in range. Researchers were interested in campers’ blood glucose levels while at camp, thus extracting the CGM data blood glucose readings outside of camp hours. While at camp, the time in range should be between 70 and 180 mg/dL.

### 2.5. Qualitative Data/Focus Groups

Researchers used an interpretivist research paradigm with a focus on understanding how youth with type 1 diabetes experience camp, specifically how they construct meaning of their chronic illness/disability (e.g., Goodwin, 2020). To support philosophical alignment, this study assumed a relativist ontology (i.e., living with diabetes is not a single, objective experience) and subjective epistemology (i.e., interpreting through the researchers’ perspective; Braun & Clarke, 2022). The research team consisted of six members, including three White women, two African-American women, and one White man. Two of the individuals have T1D. For self-reflexivity, we examined the ways in which our positionalities and previous professional and personal experiences as professional educators, camp directors, and individuals with T1D shaped our methods, interactions with campers, and data analysis. By listening to and amplifying our participants’ views about their camp experience and living with a T1D, we support youth with T1D to take an active role in sharing their views that would help shape future camp programming. 

At the conclusion of the week, staff conducted 35-min semi-structured focus group interviews with twenty-one campers with T1D (two campers missing that camp day), divided into three groups, including seven people based on length of diagnosis (less than three years, three to six years, and more than six years). Questions on the interview protocol used were grounded in the SDT and included probes, such as “what everyday decisions do you find yourself making throughout the day relating to diabetes management?” and “if you could go back in time, is there anything you would change about the choices made this week at camp?” 

After transcribing the initial recordings, focus group transcripts were coded both deductively and inductively independently by the first two authors. Interview transcripts were coded using a predefined set of codes corresponding to the research questions, followed by further investigation within emergent codes during a second and third round of coding. Throughout the coding process, proposed themes were discussed and corroborated by the two researchers (i.e., member checking). An additional research team member was employed as a critical friend to help challenge the meaning-making that was driven by the first two authors. Trustworthiness was supported by detailed notes in the form of an audit trail maintained on each step of the data analysis process. Transparency and coherence were supported throughout the research process by thoroughly describing critical elements of the research process, including data collection and analysis procedures.

## 3. Results

### 3.1. Descriptive Data

After the data were matched, 21 complete data sets (91% response rate) were analyzed using a *t*-test in SPSS V28. Fifty percent of the campers identified as male, and the average age of the campers was 12 years old. A total of 70% of campers identified as white, 16.7% as African-American, and 12.5% as Latino. The average time with diabetes among all campers was 4.5 years.

### 3.2. Quantitative Data

Of the 21 matched pairs, 11 campers used Dexcom, thus allowing camp staff to monitor their blood glucose while at camp. The overall average blood glucose level for campers was 183 mg/dL during camp hours (9 a.m.–4 p.m.). Additionally, after evaluating the data provided by our campers’ Dexcom, the 11 campers had the most optimal blood glucose average on Tuesday, 170 mg/dL. The authors also computed the daily average blood glucose and percentage of TIR for the week, during camp hours, as shown in Table 2. The decline of TIR throughout the week is likely due to the level of physical activity each day.

A paired-sample *t*-test was conducted to calculate composite scores for autonomy, relatedness, and competence. Although all three variables had an increase, there was no statistical difference from pre- to posttest. The composite score for autonomy (A) showed a slight increase from pretest (*M* = 3.86, *SD* = 0.50) to posttest (*M* = 3.95, *SD* = 0.49), with *t*(22) = −0.731, *p* = 0.15, and Cohen’s *d* = 0.23 (small). The composite score for relatedness (R) showed an increase from pretest (*M* = 4.11, *SD* = 0.73) to posttest (*M* = 4.45, *SD* = 0.59), with *t*(22) = −2.049, *p* = 0.05, and Cohen’s *d* = 0.51 (medium). The composite score for competence (C) showed a modest increase from pretest (*M* = 3.86, *SD* = 0.64) to posttest (*M* = 3.99, *SD* = 0.65), with *t*(22) = −0.903, *p* = 0.37, and Cohen’s *d* = 0.19 (small).

### 3.3. Qualitative Data

Participants in the study shared opportunities to build self-determined behaviors within camp, and perceived attitudes and perspectives related to diabetes management were described in three themes. The three themes identified were lack of outside understanding, varying levels of autonomy, and experiences at REACH. Theme one, a lack of outside understanding, revolved around society’s lack of information about T1D, making participants feel misunderstood. Additional education is needed so others are correctly informed about what is going on for a person with T1D. Participant A said “I don’t think people really get it unless they have diabetes too.” Participant B said “It’s hard to tell if I’m high or if it’s just a normal headache… people don’t understand that it feels different.” They described feeling as if others did not understand anything about the treatment or management of diabetes. Participant C said “other kids mock my insulin pump” and felt like they must disclose their T1D to everyone while explaining the truths of T1D, which others would not understand.

For theme two, varying levels of autonomy, participants expressed that their autonomy changes through the day, depending on their context or situation. For example, youth expressed that their parents would help monitor them throughout the night to treat low blood glucose values. Participant D said “I’m pretty independent with my mom. She’s trying to get me more used to doing it myself so I can go on an airline flight by myself… I can do everything myself.” During school, they felt as if their autonomy was taken away through daily situations and the school setting. Throughout sports and clubs, the youth expressed that it was easier to navigate those situations with the assistance of others. Participant E said “I do feel pretty confident about handling what I need to do… in a wild situation, like when I have three units on board before soccer, I just sit down and see what happens.” This looked like several different things: moms watching blood sugars, taking breaks, or even stopping practice when blood glucose values were above 400. Each individual had their own way of alerting others while being physically active. Participant F said “I do my corrections myself most of the time, but if it’s not going down, my mom steps in.”

For theme three, experiences at REACH, youth shared multiple opportunities to engage in activities and behaviors that promote competence, autonomy, and relatedness. The youth had the ability to explore the effects of new foods in a successful way, which they had the support of the camp staff. Participant G said “The counselors like Noah and Alistair really added extra to my camp experience.” Participants enjoyed having less parental involvement through the process of counting carbs, treating blood sugar values, and doing it on their own. Participant H said “It was nice being around people who know what it’s like.” Through the use of CampViews (an electronic medical record platform), staff were able to see the youth’s blood glucose values. This platform allowed staff to support campers’ meal choices. Participant I said “It’s really great… what other opportunities would allow you to be more independent in a setting like this?” With the camp only having youth with T1D, they expressed a sense of community and understanding in the space of REACH. Campers emphasized the value of peer and counselor relationships, noting that being surrounded by others who shared their experiences with diabetes fostered a sense of belonging and comfort. Further support of autonomy was described through REACH as a space where they could practice making independent decisions about insulin dosing, nutrition, and activity—skills often mediated by parents at home. This structured independence increased self-confidence and competence in managing diabetes, both critical components of sustained motivation.

## 4. Discussion

With the rising prevalence of T1D, the utilization of programs, such as medical specialty camps, to promote positive health outcomes should be considered, particularly for youth who may struggle to meet target A1C levels (Collins et al., 2021). Understanding the experiences of program participants can provide both healthcare professionals and medical camp staff with unique insight into opportunities that can support camper autonomy. The goal of the camp was to use Outcome-Focused Programming grounded in Self-Determination Theory (SDT) to help tween/teen participants internalize better diabetes management skills while also measuring time in range at the camp. The three basic psychological needs of autonomy, competence, and relatedness trended in a positive direction, but relatedness showed the biggest improvement. The lack of significance could be due to the short timeframe of the camp. It is possible that these scores would show a significant gain in three months due to the psychological needs (e.g., competence), taking time to show change in individuals. The competence and autonomy outcomes likely need more time, but there are new spaces for youth to demonstrate their newly acquired knowledge. Demonstrating autonomy would also require an autonomy-supportive environment for youth to exercise effective choices. This approach could be designed within parent training programs of youth with T1D in some family camps (Hill et al., 2022). The BPNSFS has not yet been used in a diabetes camp setting, adding to the body of knowledge on this topic. Additionally, focus groups with participants can provide evaluative data that provides insight into contextual influences that can contribute to participants’ overall program experience, including interactions with peers, families, and other significant individuals within their lives.

With wide support from volunteer camp medical professionals, this program has demonstrated outcomes that are providing the youth we serve with autonomy, competence, and relatedness (Blaylock et al., 2025; Collins et al., 2021). The use of CampViews to monitor campers’ blood glucose levels and their time in range while attending camp builds on current research within the field. Campers maintained an acceptable range while at camp of an average blood glucose level of 178 mg/dL. However, this is still outside the ADA-recommended target. These findings are similar to other camp studies where camp is beneficial, yet the target TIR could not be achieved despite programming efforts (Nagl et al., 2022). This data supports other research that “youth with T1D can benefit from a high level of physical activity without undue fear of hypoglycemia” (Gal et al., 2022). Monday’s camp day resulted in the highest percentage of time in range, where activities included swimming, walking, tie-dying, and outdoor field games. Monday was the most active day, and campers had the most desired TIR. Overall, this data helps to inform perspectives of youth living with T1D.

The qualitative data support the quantitative findings. The three themes, lack of outside understanding, varying levels of autonomy, and experiences at REACH, add to the current body of literature. The third theme, connecting with others at REACH, aligns with the quantitative outcome of relatedness, having the largest increase from pre- to posttest. This supports clinical significance and should continue to be evaluated to see what other spaces could support relatedness. Both the quantitative and qualitative data highlight the opportunity/importance of relatedness at camp. Youth with T1D are often the only individuals in their school; approximately 1 in 300 youth have T1D. This isolation can be highly challenging, especially during critical times of adolescent development. Camp can provide that space to help youth connect with others who face similar daily challenges. Williams et al. (2024) suggest that COVID-19 significantly impacted programs and services in the medical camp space and the choices made. The current study’s findings are consistent with other research where themes were food and insulin dosing, challenging food environments, and nutrition education, resources, and support (Blaylock et al., 2025). Finally, the current study also supports research identifying diabetes as an added responsibility of living, youth voice, and a knowledge-based community (Hill et al., 2025). Collectively, through qualitative data and additional research, it seems that youth with T1D need choice and community, both of which are attainable at diabetes camp.

### 4.1. Limitations

This study had limitations. The sample size was small, which affected the significance of the results. However, many medical specialty camps are smaller than non-diabetic peer camps. The cross-sectional, non-controlled design restricts causal inference. This was only a day camp, meaning the evening and outside of camp time could have impacted the results. Additionally, the sample size was not very diverse for a T1D study; 70% of the sample identified as White. There was also no comparison group, limiting the impact of the camp’s intention. The TIR needs more attention. We could only access half the campers’ blood glucose through CampViews. CampViews has not been validated; manual glucose verification or other comparisons could help support its use. Future studies should pursue more effective ways to gain access to all campers’ blood glucose and compare it outside of camp hours. Future studies should consider measuring the BPNFS 3, 6, or 12 months post-camp to determine if changes have taken place later as youth have time to process the traits. Finally, a potential limitation came from surveying the youth; campers may score questions by trying to please camp counselors.

### 4.2. Practitioner Implications

This OFP model, grounded in SDT and campers’ basic psychological needs, can be utilized in a wide variety of settings. Specifically, this model can be implemented in other medical specialties and traditional camps. This research is centered around campers’ motivation to manage their diabetes, but the theoretical basis of SDT can be applied to any human behavior requiring motivation (Ryan & Deci, 2020). Other camps can apply this OFP model to identify if participants’ levels of perceived autonomy, competence, and relatedness are further met after participating in the camp. Future research should investigate longitudinal studies on participants who attend multiple diabetes camp programs throughout the year to determine if campers maintain a more effective time in range while participating. The percentage of TIR is significant within the clinical setting for goal setting. Additionally, the study attempted to obtain the psycho-social and biometric outcomes of the distinctive camp on campus model serving youth with diabetes. The current diabetes camp model, aligned with other models with a focus on outdoor recreation, rather than all education, shows promise (e.g., Kietaibl et al., 2024). Other research supports the camp experience for youth with T1D as beneficial for management and positive psychosocial outcomes (e.g., Bultas et al., 2016). The current model and data contribute to building a community of practice within the diabetes camp world by way of shared interest, creating a network, and assisting practitioners to improve quality of life. 

## 5. Conclusions

With diabetes management, the individual’s motivation needs to be taken into consideration. Self-Determination Theory argues that when autonomy, competence, and relatedness are present, a person is able to internalize healthy behaviors. When youth with T1D can engage in specialized recreational opportunities grounded in SDT, they gain the support needed to internalize the behaviors needed to successfully manage their diabetes. Specifically, diabetes education is crucial to successful management, and integrating it into the camp setting allows campers to support and learn from one another (Harvey et al., 2022). This exploratory study, looking at motivation for management, percentage of TIR, and focus groups of youth living with T1D, supports previous work and adds value by connecting cloud-based technology. Although there was no statistical significance, it can be used for other programs to scale for a larger sample size and more effective engineering camp experiences. Campers did express a sense of community while at REACH, and that is due to programs only being offered to youth with T1D and their siblings or close friends (Hobbs et al., 2022). Future research should continue to capitalize on creating spaces where they are not the minority regarding their chronic condition and creating the opportunity for relatedness. Finally, medical specialty camps for youth with T1D have the potential to positively impact campers’ blood glucose levels by providing engaging activities that incorporate physical activity, advocacy, education, and healthy behaviors into campers’ daily routine (Arrington et al., 2022). Overall, camp created a safe space for youth to learn more about their diabetes from their peers, camp counselors, a dietician, and diabetes educators, all while under the direct supervision of multiple licensed healthcare professionals. The qualitative theme of experiences at REACH supports more programming, such as year-round options. This camp space also helps them realize they are not alone in the T1D world. The burden of self-care can be highly demanding for adolescents with T1D, putting an additional strain on their mental health (J. W. Gregory et al., 2022). This research continues to emphasize the need for and importance of a community of practice in which medical specialty camps, such as this one, can provide a space to try new management strategies, ask questions that may not be possible in clinical settings, and connect with peers (Blaylock et al., 2025).

## Figures and Tables

**Table 1:** Camp activities and Self-Determination Theory-based outcomes.

Self-Determination Theory Outcomes Programmed	Camp Activities	Examples of Activities Supporting Self-Determination Theory Constructs
Competence and Relatedness	Rock Climbing	Learning how climbing may affect individual blood glucose levels, encouraging and competing with peers
Autonomy	Swimming	Making choices about monitoring blood glucose levels while swimming
Relatedness	Tie Dye	Discussing their type 1 diabetes and diabetes technology with peers
Competence	Nutrition Jeopardy	Learning about nutrition and how it impacts their diabetes
Autonomy, Competence, and Relatedness	Lunch	Choosing the portion and the type of foods they eat, carb counting independently or with assistance from camp staff or peers rather than calling home, socializing with their peers and counselors
Autonomy, Competence, and Relatedness	STEM and Arts and Crafts Activities	Choosing activities, discussing how sedentary activities may affect an individual’s blood glucose levels, working in small groups to create their projects

**Table 2:** Percentage of time in range.

Day	Daily Average BG	% Time in Range
Monday	180	60%
Tuesday	170	56%
Wednesday	193	47%
Thursday	180	55%
Friday	193	49%

## Data Availability

The data presented in this study are available upon request from the corresponding author due to privacy concerns.

## Abbreviations

The following abbreviations are used in this manuscript:

SDT	Self-Determination Theory
CGM	Continuous Glucose Monitor
OFP	Outcome-Focused Programming
